# Optimizing the risk stratification of astrocytic tumors by applying the cIMPACT-NOW Update 3 signature: real-word single center experience

**DOI:** 10.1038/s41598-023-46701-z

**Published:** 2023-11-16

**Authors:** Carmen Molica, Alessio Gili, Carlotta Nardelli, Tiziana Pierini, Silvia Arniani, Donatella Beacci, Elena Mavridou, Martina Mandarano, Rodolfo Corinaldesi, Giulio Metro, Paolo Gorello, Paolo Giovenali, Nunzia Cenci, Corrado Castrioto, Marco Lupattelli, Fausto Roila, Cristina Mecucci, Roberta La Starza

**Affiliations:** 1 Medical Oncology, S. Maria Della Misericordia Hospital, Piazzale Giorgio Menghini 8/9, 06132 Perugia, Italy; 2 Public Health Section, Department of Experimental Medicine, University of Perugia, Perugia, Italy; 3 Molecular Medicine Laboratory, Centro di Ricerche Emato-Oncologiche (C.R.E.O.), S. Maria Della Misericordia Hospital, University of Perugia, Piazzale Menghini 9, 06132 Perugia, Italy; 4 Diagnostic Cytology and Histology Unit, S. Maria Della Misericordia Hospital, Piazzale Giorgio Menghini 8/9, 06132 Perugia, Italy; 5 Division of Neurosurgery, S. Maria Della Misericordia Hospital, Piazzale Giorgio Menghini 8/9, 06132 Perugia, Italy; 6 Department of Chemistry, Biology and Biotechnology, University of Perugia, 06100 Perugia, Italy; 7 Division of Radiotherapy, S. Maria Della Misericordia Hospital, Piazzale Giorgio Menghini 8/9, 06132 Perugia, Italy

**Keywords:** Cancer, Cancer genomics

## Abstract

Our work reports implementation of a useful genetic diagnosis for the clinical managment of patients with astrocytic tumors. We investigated 313 prospectively recruited diffuse astrocytic tumours by applying the cIMPACT-NOW Update 3 signature. The cIMPACT-NOW Update 3 (cIMPACT-NOW 3) markers, i.e., alterations of *TERT* promoter, *EGFR*, and/or chromosome 7 and 10, characterized 96.4% of *IDH*^wt^ cases. Interestingly, it was also found in 48,5% of *IDH*^mut^ cases. According to the genomic profile, four genetic subgroups could be distinguished: (1) *ID*^wt^/cIMPACT-NOW 3 (n = 270); (2) *IDH*^wt^/cIMPACT-NOW 3 negative (= 10); (3) *IDH*^mut^/cIMPACT-NOW 3 (n = 16); and 4) *IDH*^mut^/cIMPACT-NOW 3 negative (n = 17). Multivariate analysis confirmed that *IDH1/2* mutations confer a favorable prognosis (*IDH*^wt^, HR 2.91 95% CI 1.39–6.06), and validated the prognostic value of the cIMPACT-NOW 3 signature (cIMPACT-NOW 3, HR 2.15 95% CI 1.15–4.03). To accurately identify relevant prognostic categories, overcoming the limitations of histopathology and immunohistochemistry, molecular-cytogenetic analyses must be fully integrated into the diagnostic work-up of astrocytic tumors.

## Introduction

The evaluation and diagnostic application of molecular-cytogenetic markers, in astrocytic and oligodendroglial tumors, is rapidly evolving and sees the introduction of a relatively large number of genetic markers, in the fifth Edition of the World Health Organization (WHO) Classification for Central Nervous System Tumors (CNS)^1,2^. Starting from the work of the Consortium to Inform Molecular and Practical Approaches to CNS tumours (cIMPACT-NOW Updates), new alterations have been recognized to be of diagnostic and clinical value. Indeed, although grading has exclusively relied on histopathological features, molecular-cytogenetics have already provided useful predictive and/or prognostic information^1,3^. In addition, as different oncogenic events are associated with clinical and pathological features that define distinct entities, new tumor types and subtypes have been introduced^1^. As for gliomas, glioneuronal tumors, and neuronal tumors, these have been reclassified into six families, including "diffuse adult-type gliomas," which account for the vast majority of primary brain tumors in adults.

It is worth noting that *EGFR* amplification, gain of chromosome 7, monosomy 10, *PTEN* mono- or bi- allelic deletions, and mutations of *TERT* promoter (*TERT*p), typically occur in grade IV, i.e. glioblastoma (GBM), and in *IDH1/2*-wildtype (*IDH*^wt^) grade II/III, i.e. diffuse/anaplastic astrocytomas (DA/AA), where their prognostic impact is still debated^4–6^. In 2018 Stichel et al. first proposed the use of these markers as prognostic factors to improve patient risk assignment^7^. Afterwards, the cIMPACT-NOW Update 3 consortium, has recommended their employment to define a high risk signature, valuable to fine tune the classification of *IDH*^wt^ DA/AA^8^. Accordingly, cases harboring *EGFR* amplification, and/or gain of chromosome 7 plus monosomy 10 (+ 7/− 10), and/or *TERT*p mutations, have to be referred to as “astrocytic gliomas, *IDH*^wt^, with molecular features of GBM grade IV”^8^. This signature would overtake histopathology and immunohistochemistry, deeply modifying the currently adopted risk stratification criteria for *IDH*^wt^ DA/AA^9^. However, implementation of these bio-molecular criteria for routine diagnostics of glioma tumors is still not widely applied.

Starting from our "real-life" experience, we sought to assess the correlation between genomic profile and disease outcome in adult patients with glial tumors based on data obtained from the application of a multidisciplinary diagnostic approach that included cytogenetic and molecular characterization. Our data set on 313 cases confirmed the favorable prognostic value of *IDH1/2* mutations, and validated the cIMPACT-NOW 3 signature as high-risk marker, thus confirming prognostically relevant subgroups.

## Materials and methods

### Patient cohort

All patients prospectively admitted to the Neurosurgery department of our Regional Hospital and referred to the Molecular Medicine Laboratory (Department of Medicine and Surgery, University of Perugia), for molecular-cytogenetic diagnosis, were included in the study (timeframe: from August 2010 to July 2020). Overall, 313 patients were recruited. There were 184 males and 129 females. The median age, at diagnosis, was 64 years (range 23–83); the median follow-up was 10.9 with a range of 0.2–168 months. Survival was measured from the date of histopathological diagnosis until death, or was censored at the date of last follow-up. Median follow-up was 18.3 months: 24.3 for DA/AA and 9.6 for GBM.

In all cases the backbone of treatment was based on alkylating agents and/or radiotherapy except for 41 patients with multifocal diffuse disease and compromised clinical conditions, who died within 3 months from diagnosis. Timing, dosing, and scheduling were determined based on age, Karnofsky performance status, size of residual tumour, and presence of multifocal lesions. Radiotherapy started within 3–5 weeks after surgery, at a dosage of 50–60 Gy in 1.8–2 Gy, daily fractions. In fit patients, aged less than 70 years, temozolomide was concomitantly administered at 75 mg/m^2^ daily dosage, plus at least six cycles of maintenance (150–200 mg/m^2^, 5 out of 28 days)^10^. Patients with DA/AA were treated with radiotherapy alone or in combination with alkylating agents (temozolomide, lomustine, or carmustine)^11,12^. Molecular cytogenetic studies were done on paraffin-embedded biopsies taken at the time of diagnosis. Analyses were performed on representative areas as indicated by Pathologists.

### Fluorescence in situ hybridization (FISH)

Numerical and structural chromosome abnormalities were investigated by FISH on 4 μm Formalin-Fixed Paraffin Embedded (FFPE) cerebral tissue sections, after automated chemical pretreatment by ThermoBrite Elite (Leica, Milan, Italy). Commercially available genomic probes were applied to study EGFR amplification and chromosome 7 numerical abnormalities (LSI EGFR Spectrum orange/CEP 7 Spectrum green probe, Vysis-Abbott Milan, Italy), PTEN deletions and chromosome 10 numerical abnormalities (LSI PTEN Spectrum orange/CEP 10 Spectrum green probe, Vysis-Abbott), and codeletion of 1p and19q (codel1p/19q) (LSI 1p36 SpectrumOrange/1q25 SpectrumGreen Probes and s LSI 19q13 SpectrumOrange/19p13 SpectrumGreen Probes, Vysis-Abbott). Analyses were carried out on areas with > 50% of infiltrating neoplastic cells, using an Olympus BX61 fluorescence microscopy (Olympus, Milan, Italy) equipped with a highly sensitive JAI camera (Copenhagen, Denmark) and driven by CytoVision 4.5.4 software (Genetix, New Milton, Hampshire, UK). To avoid missing of small abnormal clones, microscopic analysis of the entire hybridization area (18 × 18), and evaluation of at least 200 cells, was performed. Cut-off for analyses were set as follows: *EGFR* amplification ≥ 3% of analyzed cells, chromosome 7 gain ≥ 10%, *PTEN* deletion/monosomy 10 ≥ 20%^13^.

### Sanger sequencing

Genomic DNA, extracted from 8 μm FFPE brain tumor sections using an automatized Qiacube system and the QIAamp DNA FFPE Tissue Kit (Qiagen, Milan, Italy) and investigated for IDH1 (exon 4, 385 bp), IDH2 (exon 4, 312 bp), and core TERT promoter (TERTp) hot-spot mutations by ABI 3500 Genetic analyzer instrument (Applied Biosystems, Monza, Italy). The primer design has already been reported by Pierini et al.^13^ and was referred to the GRCh37 genomic coordinate system: NM_005896.3 for *IDH1*, NM_002168.3 for *IDH2*, and NM_000005.9 for core TERTp. Sequence analysis was performed with EditSeq DNAstar and FinchTV1 software, and alignment was supported by Clustal Omega (http://www.ebi.ac.uk/Tools/msa/clustalo), and the analysis of variants by Ensembl (http://www.ensembl.org/Homo_sapiens), Varsome (https://varsome.com), and COSMIC (https://cancer.sanger.ac.uk/cosmic) websites.

### Statistical analysis

Descriptive statistics were calculated including frequencies, percentages, frequency tables for categorical variables, median and means ± standard deviation (SD) for quantitative variables. Categorical variables were evaluated by Chi-square or Fisher's exact test when appropriate. The Kaplan–Meier method was used to analyze Overall survival (OS) and estimate medians with two-sided 95% confidence intervals (CI). Survival curves were compared using the log-rank test. Cox regression model was applied to estimate Hazard Ratio (HR) and 95% CI and to identify prognostic factors independently associated with survival times. To test proportional hazard (PH) assumption log-minus-log plots was used^14^. Stepwise backward-selection was used for eliminates variables from the regression model to find a reduced model that best explains the data^15^. A p-value of less than 0.05 was considered to be statistically significant. Statistical analyses were performed with STATA v. 16.1 (StataCorp LP, College Station, TX, USA).

### Ethics aproval and consent to particpate

This study was performed in line with the principles of the Declaration of Helsinki and it was approved by the Umbria region ethic committee, code number 2843/16 on August 8th, 2016. Informed consent, regarding data was obtained from all individual participants included in the study.

## Results

### The genomic profile of glioma tumours

Cases were first classified on the basis of histopathological and immunohistochemistry features as GBM (276), AA (17), and DA (20) (Table 1)^2^. According to the status of *IDH1/2* genes, there were 280 *IDH* wildtype (*IDH*^wt^) cases, including 267 GBM, 8 DA, and 5 AA, and 29 *IDH-*mutant (*IDH*^mut^), which harbored *IDH1*R132, *IDH2*R140, or *IDH2*R172^1^. They were 5 GBM, 12 DA, and 12 AA (Table 1). In addition, 4 cases had non-canonical *IDH1* or *IDH2* mutations (cases nos. 3, 4, 6, and 9, Table 2). These mutations were all located within the hot-spot region (exon 4), were not reported as polymorphisms, and were predicted to be pathogenic/likely pathogenic and/or described as somatic in other tumor types (Table 2)^16–19^. Therefore, since cases with non-canonical *IDH1/IDH2* mutations could not be definitively considered *IDH*^wt^, we grouped them with *IDH*^mut^ cases, while not strictly applying the WHO criteria^1^. Altogether, in our case series a total of 33 cases were considered to be *IDH*^mut^.

**Table 1:** Distribution of *IDH1/2* mutations and cIMPACT-NOW 3 alterations in histological subgroups.

WHO (2016)	DA	AA	GBM	Overall
*IDH*^wt^	8	5	267	280
*IDH*^mut^	12	12	9	33
*TERT*p^mut^	7	6	237	250
*EGFR* amplification	4	2	105	111
Gain chromosome 7	13	14	147	174
Monosomy 10	8	5	196	209
*PTEN* deletion	0	3	16	19
+ 7/-10	2	4	116	122

WHO, World Health Organization; DA, diffuse astrocytoma; AA, anaplastic astrocytoma; GBM glioblastoma; *IDH*, refers to both *IDH1* and *IDH2*; wt, wild type; mut, mutation; + 7, multiple copies of chromosome 7; -10, monosomy 10.

**Table 2:** Clinical and molecular-cytogenetic features of 12 *IDH*^mut^ glioma cases harboring the cIMPACT-NOW 3 signature.

Cases	Overall survival	Status	S	A	Istopathology (WHO 2016)	*IDH1*	*IDH2*	*TERT*p	*EGFR*	+ 7/− 10
1	9.34	Alive	F	45	DA *IDH*^mut^	c.395 G>A (p.R132H)	wt	wt	ampl	No
2	2.87	Dead	M	78	DA *IDH*^mut^	c.395G>A (p.R132H) rs11554137C>T	wt	c.1–124 C>T (g.1295228)	wt	No
3	1.66	Dead	M	57	GBM, *IDH*^mut^	wt	c.497 A>G (p.K166R)	c.1–124 C>T (g.1295228 C>T)	ampl	No
4	0.82	Alive	M	81	GBM, *IDH*^mut^	c.253 G>A (p.E85K)	wt	wt	wt	Yes
5	4.84	Alive	F	70	DA *IDH*^mut^	c.395 G>A (p.R132H)	wt	wt	wt	Yes
6	2.25	Dead	F	66	GBM, *IDH*^mut^	wt	c.528 C>T (p.G176G)	c.1-124C>T (g.1295228)	wt	Yes
7	4.6	Alive	M	43	AA *IDH*^mut^	c.395 G>A (p.R132H)	wt	c.1–124 C>T (g.1295228)	wt	No
8	11.83	Alive	F	53	AA *IDH*^mut^	c.394 C>T (p.R132C)	wt	wt	wt	Yes
9	0.23	Dead	F	67	GBM, *IDH*^mut^	c.210C>T (p.G70G)	wt	c.1–146 C>T (g.1295250 C>T)	wt	Yes
10	1.17	Dead	M	74	GBM, *IDH*^mut^	wt	c.419 G>A (p.R140Q)	c.1-124C>T (g.1295228)	ampl	No
11	3.43	Alive	M	59	GBM, *IDH*^mut^	c.395 G>A (p.R132H)	wt	wt	wt	Yes
12	2.18	Alive	F	41	GBM, *IDH*^mut^	c.394 C>G (p.R132G)	wt	wt	wt	Yes
13	1.68	Alive	M	54	AA *IDH*^mut^	c.395 G>T (p.R132L) rs11554137 C>T	wt	wt	wt	Yes
14	1.51	Alive	M	31	AA *IDH*^mut^	c.395 G>A (p.R132H)	wt	wt	wt	Yes
15	0.73	Alive	F	49	DA *IDH*^mut^	c.395 G>A (p.R132H)	wt	wt	wt	Yes
16	0.21	Alive	F	50	AA *IDH*^mut^	c.395 G>A (p.R132H)	wt	wt	wt	Yes

Cases Nos. 3, 4, 6, and 9 have non-canonical *IDH* mutations that do not meet the WHO fifth classification criteria for defining *IDH*-mutant cases.

Non-canonical *IDH1* variants: c.253G>A (p.E85K), (https://varsome.com; Uson Junior PLS, et al., Cancer Manag Res.^17^); c.210C>T (p.G70G), (Giannakis et al., Cell Rep.^16^).

Non-canonical *IDH2* variants: c.497 A>G (p.K166R), variant of uncertain significance (not reported as a polymorphism; gnomAD database); c.528 C>T (p.G176G) (Mouradov et al., Cancer Res.^19^).

*S* sex, *A* age, *M* male, *F* female, *DA* diffuse astrocytoma, *GBM* glioblastoma, *AA* anaplastic astrocytoma, *wt* wild type, *mut* mutation, *ampl* amplification.

Overall, *EGFR* amplification (*EGFR* ampl) was detected in 111/306, gain of chromosome 7 in 174/306, and *TERT*p mutations in 250/313. Monosomy of chromosome 10 was found in 209/300 cases while mono- and/or bi- allelic deletion of *PTEN* in 19/300. We observed that high risk molecular-cytogenetic markers were unequally distributed among GBM and DA/AA. In GBM, *EGFR* ampl was detected in 105/276, monosomy of chromosome 10/*PTEN* deletion in 212/276, gain of chromosome 7 in 147/276, *TERT*p mutations in 237/276; in DA and AA, *EGFR* ampl was found in 6/37 (4/20 DA and 2/17 AA), gain of chromosome 7 in 27/37 (13/20 DA and 14/17 AA), monosomy of chromosome 10/*PTEN* deletion in 16/36 (8/19 DA and 8/17 AA), *TERT*p mutations in 13/37 (7/20 DA and 6/17 AA) cases.

By applying the cIMPACT-NOW Update 3 signature, 270 cases were reclassified as “Diffuse astrocytic glioma, *IDH*-wt, with molecular features of glioblastoma, WHO grade IV” due to the presence of at least one of biomolecular markers that define the cIMPACT-NOW 3 signature. All 13 *IDH*wt DA/AA cases, 11 of whom had at least 2 high-risk cIMPACT-NOW markers and none had an isolated *TERT*p mutation, belonged to this subgroup.

The cIMPACT-NOW 3 signature also characterized 16/33 *IDH*^mut^ cases (4 DA, 5 AA and 7 GBM) (Table 2). There were 9 cases with + 7/− 10, 1 case with *EGFR* amplification, and 2 cases with *TERT*p mutations. In the remaining 4 cases, 2 concomitant high-risk markers were detected: + 7/− 10 and *TERT*p mutations (= 2) or *EGFR* amplification and *TERT*p mutations (= 2). In all cases, the absence of codel1p/19q excluded the diagnosis of oligodeldroglioma^1,2,20,21^. On the other hand, the cIMPACT-NOW 3 signature was not found in 10 patients with *IDH1*^wt^ GBM (Table 3) showing the typical morphology of diffuse astrocytic tumors, with microvascular proliferation and/or palisade necrosis^1,2^.

**Table3:** Main features of *IDH*^wt^ GBM without the cIMPACT NOW 3 signature.

Case	Sex	Age	Diagnosis (WHO 2016)	Follow-up (years)	Status
1	F	63	GBM *IDH*^wt^	4.23	Died
2	F	48	GBM *IDH*^wt^	3.98	Died
3	M	51	GBM *IDH*^wt^	5.40	Alive
4	M	45	GBM *IDH*^wt^	0.05	Died
5	M	71	GBM *IDH*^wt^	5.29	Alive
6	F	77	GBM *IDH*^wt^	0.85	Died
7	F	65	GBM *IDH*^wt^	8.38	Alive
8	F	71	GBM *IDH*^wt^	8.05	Alive
9	F	67	GBM *IDH*^wt^	0.43	Died
10	M	12	GBM *IDH*^wt^	2.46	Died

*F* female, *M* male, *wt* wild type.

### Genomic features and survival

**Figure 1:**
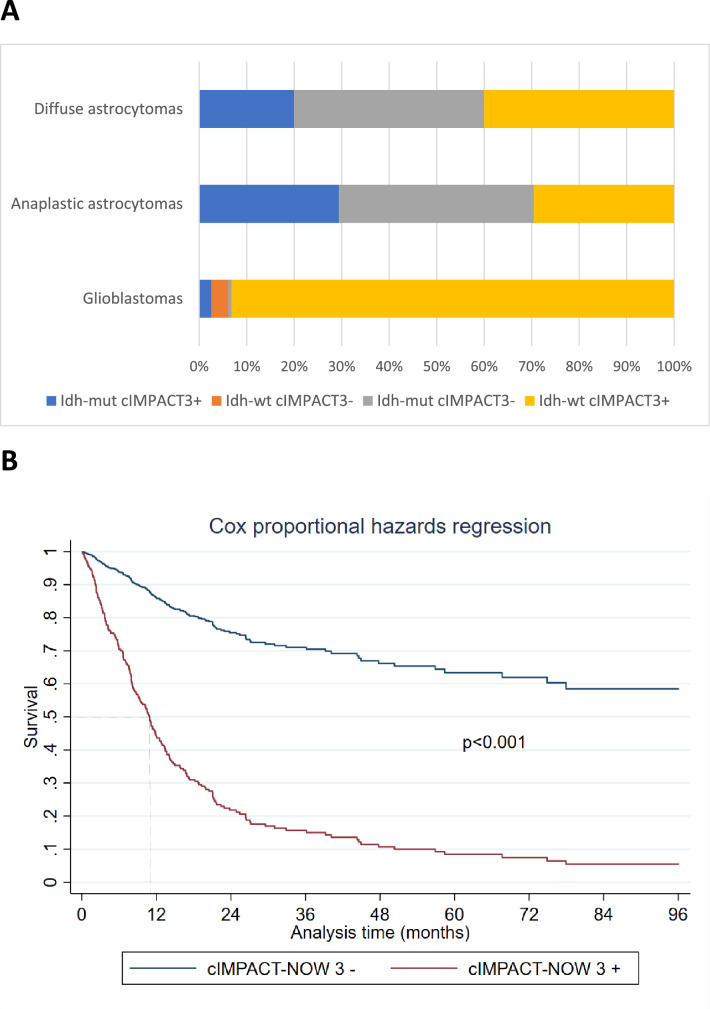
(**A**) Distribution of *IDH1/2* mutations and cIMPACT-NOW 3 markers according to histopathology; (**B**) Estimated overall survival after fitting Cox model by cIMPACT-NOW 3 signature.

According to genetic profile the entire cohort of cases was re-classified in four subgroups: (1) *IDH*^wt^/cIMPACT 3 (= 270); (2) *IDH*^wt^/cIMPACT 3 negative (= 10); (3) *IDH*^mut^/cIMPACT 3 negative (= 17); and (4) *IDH*^mut^/cIMPACT 3 (= 16) (Fig. 1A). While the cIMPACT 3 signature, the *IDH* status and age, were all strong predictors of outcome, histopathology and immunohistochemistry lost their prognostic significance.

The *IDH* status was confirmed to be a robust prognostic marker (*IDH*^wt^, HR 2.91 95% CI 1.39–6.06) distinguishing two relevant risk subgroups. We also validated the cIMPACT-NOW 3 signature as an additional prognostic marker which improved the stratification of *IDH*^wt^ tumours (cIMPACT-NOW 3, HR 2.15 95% CI 1.15–4.03). Indeed, the probability of overall survival was significantly lower in cIMPACT-NOW 3 positive (high risk subgroup) than in *IDH*^wt^ cIMPACT-NOW 3 negative tumours (low risk subgroup) (Fig. 1B).

The 13 *IDH*^wt^ DA/AA cases that belonged to the cIMPACT-NOW 3 high risk subgroup showed a median overall survival (8.8 months; range 10–47) similar to GBM cases, although the two medians were statistically different probably due to the limited number of these rare cases. In addition, 12/13 patients died from disease progression. Instead, the cIMPACT-NOW 3 signature did not appear to be prognostically relevant in *IDH*^mut^ cases (Table 2). On the other hand, according to the overall survival, the cIMPACT-NOW 3 negative GBM cases, either *IDH*^wt^ (Table 3) or *IDH*^mut^, could be probably relocated into a more favorable risk subgroup (median overall survival: 55.8 months (29.5-not reached).

## Discussion

Our study, conducted on a prospectively recruited case series, has the strength to broadly represent the incidence and distribution of recurrent astrocytic tumors in adult subjects in the real world. Indeed, as expected in adults, the number of patients with low grade *IDH*^mut^ tumors was rather low. Thus, we are aware that this type of study might cause a bias in the analysis of patient survival related to differences in treatment and underrepresentation of rare tumor subtypes. On the other hand, the study precisely reflects the epidemiological features of astrocytic tumors in adults. In addition, as a long-term follow-up was available, the outcome of long-term survivors could be precisely determined. Overall, results from our study further support the rationale for incorporating the cIMPACT-NOW 3 signature as diagnostic criteria for glioblastoma^1^.

We confirmed that both *IDH* status and cIMPACT-NOW 3 signature are valid prognostic markers for risk stratification of patients and also provided new insights to consider for reclassification of low-risk cases.

As expected, the *IDH* status was found to be a robust prognostic marker distinguishing two relevant risk subgroups. Then to determine the impact of the genomic profile on overall survival, we assessed how the cIMPACT-NOW 3 signature was distributed into *IDH*^wt^ and *IDH*^mut^ cases. As expected, the large majority of *IDH*^wt^ cases were characterized by high risk signature. In particular, 92.6% cases were positive for at least two markers, and *TERT*p mutations were detected in 90% cases. Interestingly, 10 (~ 3.5%) *IDH*^wt^ GBM did not harbor the cIMPACT-NOW 3 signature.

Survival analysis clearly indicated that the cIMPACT-NOW 3 signature is an additional prognostic marker suitable to improve the risk stratification of *IDH*^wt^ cases. In fact, the probability of overall survival was significantly lower in cases with the cIMPACT-NOW 3 signature than in cases without this high-risk marker.. Notably, all the 13 *IDH*^wt^ DA/AA belonged to the cIMPACT-NOW 3 high risk subgroup and showed a median overall survival similar to GBM cases. This is probably due to the genomic profile of our *IDH*wt DA/AA cases, which never had the isolated *TERT*p variants and showed 2–3 high-risk markers. Similarly, Berzero et al.^22^ reported that astrocytomas with 2–3 molecular traits of the cIMPACT-NOW 3 signature had a more severe prognosis. However, they also showed that high risk markers are unequally distributed among grade II and grade III astrocytoma and that isolated TERTp mutations were not predictive of poor outcome^22^.

Other groups have shown that glioma cases with gain of 7p, loss of 10q, and mutation in the TERT promoter biologically represent a different subtype, which is prognostically relevant and has clinical impact for proper risk stratification of patients and choice of treatment^23–25^. On the other hand, we and others have observed that *IDH*wt GBM without the cIMPACT-NOW 3 signature behaved as low risk gliomas showing a long survival.

These findings, based on data from real-world diagnostic activity, further highlight the inherent limitations of histopathology and immunohistochemistry in the classification of glial tumors, and indicate the need for comprehensive characterization to properly stratify patients at the time of diagnosis. They are in line with other studies that have shown that *IDH*^wt^ astrocytomas are more likely to behave like GBM, even in the absence of a high-risk molecular profile^24,26^. However, to confirm these data, large multicenter prospective studies need to be conducted.

Thus, while the cIMPACT-NOW 3 signature, the *IDH* status and age, were all strong predictors of outcome, histopathology and immunohistochemistry lost their prognostic significance. By introducing genetics among the dignostic criteria, the fifth edition of the WHO classification^1^ established that regardless of histopathology, cases with high-risk molecular markers should be classified as GBM *IDH*wt. Instead, according to the overall survival, GBM cases, either *IDH*^wt^ or *IDH*^mut^, not harboring this high risk signature, could be relocated into a more favorable risk subgroup.

Although the cIMPACT-NOW 3 signature was recommended to refine the classification of *IDH*^wt^ gliomas, we sought to assess whether it also occurred in cases with *IDH1/2* mutations and if it impacts upon prognosis in this subgroup of gliomas. Unexpectedly, roughly 48% of *IDH*^mut^ cases harbored cIMPACT-NOW 3 markers which however did not appear to be prognostically relevant in this subgroup of tumors.

In conclusion, as recommended by the last updated WHO classification for CNS tumors, to accurately identify relevant prognostic categories, overcoming the limitations of histopathology and immunohistochemistry, a comprehensive molecular-cytogenetic approach must be considered in the diagnostic work-up of this subgroup of human cancers. Remarkably, more than 20 genes/pathways have been proposed to refine the classification of specific nosological entities in the context of gliomas and astrocytic gliomas in children and adults^1^. Defining the genomic profile of glioma tumours is not only required to predict response to chemo-/radio- therapy and life expectancy of patients, but also to provide the molecular basis for tailored treatments.

## Data Availability

The datasets generated and analyzed during the current study are not available in the repository, but will be provided in excel format if requested, at any moment, at email adress alessio.gili@unipg.it.
